# Ethylene Is Crucial in Abscisic Acid-Mediated Modulation of Seed Vigor, Growth, and Photosynthesis of Salt-Treated Mustard

**DOI:** 10.3390/plants13162307

**Published:** 2024-08-19

**Authors:** Asim Masood, Sheen Khan, Iqbal R. Mir, Naser A. Anjum, Faisal Rasheed, Abdulrahman Al-Hashimi, Nafees A. Khan

**Affiliations:** 1 Plant Physiology and Biochemistry Laboratory, Department of Botany, Aligarh Muslim University, Aligarh 202002, India; 2 Department of Botany and Microbiology, College of Science, King Saud University, Riyadh 11451, Saudi Arabia; aalhashimi@ksu.edu.sa

**Keywords:** abscisic acid, ethylene, photosynthesis, S and N assimilation, salinity, mustard

## Abstract

The current study explored the differential interaction between ethylene (ET) and abscisic acid (ABA) in relation to salt stress in mustard (*Brassica juncea* L.) plants. Significant reductions in seed germination, growth, and photosynthesis were observed with 100 mmol NaCl. Among the cultivars tested, the Pusa Vijay cultivar was noted as ET-sensitive. Pusa Vijay responded maximally to an application of 2.0 mmol ethephon (Eth; 2-chloethyl phosphonic acid-ethylene source), and exhibited the greatest growth, photosynthesis, activity of 1-aminocyclopropane carboxylic acid (ACC) synthase (ACS), and ET evolution. Notably, Eth (2.0 mmol) more significantly improved the seed germination percentage, germination and vigor index, amylase activity, and reduced H_2_O_2_ content under salt stress, while ABA (25 µmol) had negative effects. Moreover, the individual application of Eth and ABA on Pusa Vijay under both optimal and salt-stressed conditions increased the growth and photosynthetic attributes, nitrogen (N) and sulfur (S) assimilation, and antioxidant defense machinery. The addition of aminoethoxyvinylglycine (0.01 µmol AVG, ET biosynthesis inhibitor) to ABA + NaCl-treated plants further added to the effects of ABA on parameters related to seed germination and resulted in less effectiveness of growth and photosynthesis. In contrast, the effects of Eth were seen with the addition of fluoridone (25 µmol Flu, ABA biosynthesis inhibitor) to Eth + NaCl. Thus, it can be suggested that ET is crucial for alleviating salt-induced inhibition in seed germination, growth, and photosynthesis, while ABA collaborated with ET to offer protection by regulating nutrient assimilation and enhancing antioxidant metabolism. These findings provide insight into the complex regulatory processes involved in ET–ABA interaction, enhancing our understanding of plant growth and development and the mitigation of salt stress in mustard. It opens pathways for developing hormonal-based strategies to improve crop productivity and resilience, ultimately benefiting agricultural practices amidst a challenging environment.

## 1. Introduction

Soil salinity, which affects more than 800 million hectares of land worldwide and more than 6% of the planet’s arable land, has been determined to have a significant negative impact on global crop production [1,2]. Salinization of arable land is anticipated to increase from 10% and might lead to a more than 50% decline in the average yields of important crops [3]. In recent decades, apart from the natural salinity, salinization of soils because of intensive agriculture and irrigation has also been turning into a major problem and noteworthy issue in agriculture [4,5]. However, in plants, the nature of the damage due to a high concentration of NaCl is complex, causing both hyperosmotic stress and ionic toxicity [6,7]. This leads to poor water uptake by roots and altered Na^+^, K^+^, and Cl^–^ homeostasis, resulting in ion-specific stress responses [6,8]. The high salinity in plants causes the production of reactive oxygen species (ROS) like singlet oxygen (^1^O_2_), superoxide radical (O_2_**^−^**), hydrogen peroxide (H_2_O_2_), and hydroxyl radical (OH), leading to damage to cellular membranes, proteins, nucleic acids, and photosynthetic functions, which impairs plant physiological systems and significantly reduces crop growth, yield, and agricultural output [9,10,11,12].

Despite progress in research on tolerance strategies for crop productivity in hostile environments, traditional breeding methods and genetic engineering have not sufficiently met the need for a 60% increase in global crop production by 2050 [13]. Researchers focus on understanding and manipulating plant tolerance mechanisms to maximize yield under various environmental conditions.

The signaling molecule ethylene (ET) is a crucial modulator of plant functions at the cellular, molecular, and systemic levels, involved in almost every phase of the plant life cycle, including germination, senescence, and various biological processes [14,15,16]. It influences photosynthesis directly by altering Rubisco activity and indirectly by affecting stomatal movement through interactions with other plant growth regulators [7,17,18]. Ethylene’s role in alleviating stresses like salt stress has been extensively studied [5,7,8,17], and it helps plants respond to environmental factors by increasing antioxidant enzyme levels, nutrient metabolism, and glutathione levels [19,20].

Over the years, evidence has shown that abscisic acid (ABA) modulates plant development and stress responses [21,22]. In response to environmental challenges like drought, high salinity, and temperature extremes, plants produce more ABA, activating stress-related genes for water retention through stomatal closure [23,24]. ABA reduces transpiration by regulating stomatal activity [24] and influences root growth under osmotic stress via a hormonal network with cytokinin, ET, and auxin [25].

ABA and ET interact negatively, with ET controlling guard cell signaling [26]. ABA pathway mutants (*aba1*, *aba2*, *abi1*, *abi2*) and ET signaling mutants (*etr1*, *ein2*, *ein3*) antagonistically regulate the expression of defense and stress-responsive genes [27,28,29]. In mustard under salt stress, Fatma et al. [30] demonstrated that both ethephon (Eth; 2-chloethyl phosphonic acid-ethylene source)) and sulfur (S) influenced ABA content and stomatal regulation. ET is reported to induce stomatal opening in some species and inhibit ABA-induced stomatal closure [31,32]. According to Wang and Song [33], the ET receptor ETR1 is crucial for controlling stomatal function and mediating H_2_O_2_ signaling. When ETR1 binds to ET, it blocks the H_2_O_2_ signal, thereby decreasing the stomatal response to ABA. ET-deficient and ET-insensitive mutants were reported to trigger ABA biosynthesis and responses [34,35]. ET and ABA were found to act together to control the regulation of stomatal closure [36]. Various studies, therefore, have been carried out to understand the crosstalk between ET and ABA signaling [34,37]. This study aims to select the mustard cultivar most sensitive to ET and comprehensively investigate the mechanisms of action of ET and ABA under salt stress conditions using aminoethoxyvinylglycine (AVG) and fluoridone (Flu), which are ethylene and ABA inhibitors, respectively. Specifically, it examines their impacts on seed germination, growth, photosynthetic performance, stomatal movement, nitrogen (N) and S content, and antioxidant metabolism in the cultivar most sensitive to ET. The research focuses on exploring how ET and ABA interact to modulate mustard plant responses to salt stress, providing insights to enhance crop resilience and productivity in saline environments.

## 2. Results

### 2.1. Screening of Cultivars for ET Sensitivity by Measuring Growth, Photosynthesis, 1-Aminocyclopropane-1-Carboxylic Acid (ACC) Synthase (ACS) Activity, and Ethylene Evolution

Five mustard cultivars—Pusa Vijay, Pusa Jagannath, Pusa Agrani, PM-27, and PM-28—were examined for their responses to growth and photosynthetic characteristics, ACS activity, and ET evolution. This was done to identify an ET-sensitive cultivar.

All the cultivars responded differently to Eth treatment. Eth at 2.0 mmol maximally increased plant dry mass and leaf area in Pusa Vijay (Table 1). Following the application of Eth treatments, the photosynthetic parameters, viz., photosynthetic rate (Pn), stomatal conductance (gs), and intercellular CO_2_ (Ci), increased in all five mustard cultivars. The increasing Eth concentrations enhanced Pn, gs, and Ci; however, the 2.0 mmol Eth caused the greatest rise in all cultivars. The treatment with 2.0 mmol Eth maximally increased Pn, gs, and Ci in Puse Vijay by 59.0%, 50.1%, and 58.4%, respectively, compared with the control (Table 1). The application of 2.0 mmol Eth resulted in the maximal increase in leaf ACS activity and ET evolution in all the cultivars. Among the cultivars, Pusa Vijay showed the greatest ET evolution and leaf ACS activity. ACS activity increased by 8.1%, 12.8%, and 26.8%, and ET evolution by 9.4%, 21.7%, and 38.0%, with 50, 100, and 2.0 mmol Eth treatments, respectively, in comparison with the control plants (Table 1).

### 2.2. Effect of Eth, ABA, Flu, or AVG on Seed Germination of Mustard Grown under Salt Stress

The percent germination, germination index, vigor index, and activity of amylase was markedly suppressed with NaCl (Table 2). However, there was a considerable surge in the H_2_O_2_ content and activity of ascorbate peroxidase (APX) and glutathione (GR).

The application of Eth promoted the seed germination parameters, viz., germination percentage, germination index, vigor index, and amylase activity, by 13.8%, 16.9%, 12.4%, and 28.1%, respectively, compared with the control. However, an application of 25 µmol ABA resulted in a decline in the above parameters, indicating the inhibitory role of ABA on seed germination. In comparison with the plants treated with NaCl, Eth treatment on NaCl-stressed plants increased the germination percentage by 32.6%, germination index by 35.2%, vigor index by 22.2%, and amylase activity by 49.4%. However, ABA treatment under the NaCl-stressed conditions reduced the above parameters to lower than the NaCl-treated plants (Table 2). The application of AVG to plants treated with ABA and NaCl showed the maximum reduction in the abovementioned parameters. The combined treatment of Eth + NaCl + Flu increased the seed germination attributes compared with Eth + NaCl treatment. Furthermore, under salt stress, seeds treated individually with Eth and ABA exhibited an increase in the activity of APX (218.6%, 106.5%) and GR (103.0%, 60.6%), with a considerable reduction in H_2_O_2_ content (−72.9, −63.8%), compared with the salt-stressed seedlings. Moreover, on the application of the inhibitors, a reduction in the above parameters was observed, but the maximum reduction was observed in ABA + NaCl + AVG treatment relative to Eth + NaCl + Flu. (Table 2).

### 2.3. Effect of Eth, ABA, Flu, or AVG on Growth and Photosynthesis in Mustard Grown under Salt Stress

Salt stress treatment reduced the plant dry mass and leaf area by 45.4% and 51.8%, respectively, compared with the control plants (Table 3). The application of Eth conspicuously increased these growth parameters more than did ABA treatment. The application of Eth and ABA individually under salt stress conditions increased plant dry mass (by 122.7% and 111.3%) and leaf area (by 148.7% and 130.2%) in comparison with NaCl-treated plants. Additionally, the effects of ABA-induced relief of salt stress on growth were reversed when AVG was applied to salt-stressed plants. However, no similar reversal in growth was seen when Flu was applied to the Eth-treated salt-stressed plants. (Table 3).

A pronounced decline in photosynthetic traits was noted in the plants grown under salt stress (Table 3). The application of Eth resulted in increased photosynthetic characteristics compared with their respective controls. The application of ABA also increased the above parameters, but at a slower rate than Eth application. Additionally, under stressed conditions, the separate treatment of Eth and ABA increased Pn (by 212.1% and 184.4%), gs (by 80.3% and 68.6%) Ci (by 88.5% and 72.5%), Fv/Fm (by 102.3% and 76.2%), and Rubisco activity (by 278.1% and 256.2%) in comparison with the NaCl-treated plants. (Table 3). In comparison with the plants treated with ABA + NaCl, the Pn, gs, Ci, Fv/Fm, and Rubisco activity decreased by 58.7%, 35.7%, 38.0%, 39.1%, and 63.1%, respectively, after ABA and NaCl-treated plants were given the AVG treatment. This was due to the reversal of the ABA-induced alleviation in the above parameters. However, it was observed that when Flu was applied to plants treated with Eth and NaCl, there was a reduction in the above parameters, although not to the same extent as observed when AVG was applied (Table 3).

From these findings, it is evident that both Eth and ABA are involved in alleviating salt-induced photosynthetic inhibition. Moreover, the results from the use of Eth and ABA inhibitors indicate that Eth is more effective than ABA at relieving salt-induced inhibition in photosynthesis.

### 2.4. Effect of Eth, ABA, Flu, or AVG on the Content of Na^+^ and Cl^−^ ions, H_2_O_2_, and Thiobarbituric Acid Reactive Substances (TBARS) in Mustard Grown under Salt Stress

**Figure 1:**
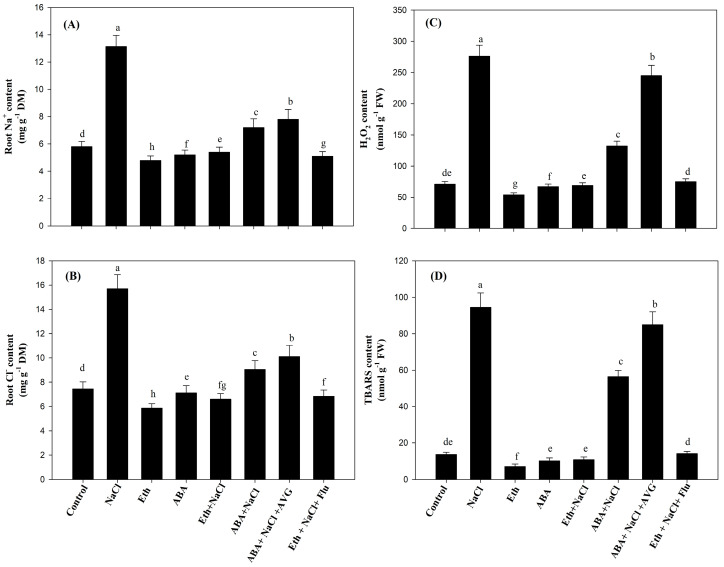
Content of root Na^+^ (**A**), Cl^−^ (**B**), H_2_O_2_ (**C**), and TBARS (**D**) in mustard (*Brassica juncea* L. cv. Pusa Vijay) seedlings grown under normal and 100 mmol NaCl stress conditions at 30 days after sowing, after foliar treatment of 2.0 mmol Eth, 25 µmol ABA, 25 µmol Flu, or 0.01 µmol AVG at 15 days after sowing. Values are presented as means ± SE (n = 4). Data means denoted by the same letter are not significantly different at *p* ≤ 0.05 according to the LSD test. ABA: abscisic acid; AVG: aminoethoxyvinylglycine; Eth: ethephon; Flu: fluoridone; H_2_O_2_: hydrogen peroxide; TBARS: thiobarbituric acid reactive substances.

Root Na^+^ and Cl^−^ ion content increased significantly, by 126.5% and 34.2%, respectively, under salt stress in comparison with the control plants. Application of Eth or ABA under salt stress reduced the content of Na^+^ by 58.9% and 45.2%, and Cl^−^ by 57.9% and 42.3%, respectively, compared with salt-stressed plants (Figure 1A,B). Plants treated with ABA + NaCl + AVG exhibited greater Na^+^ and Cl^−^ accumulations in comparison with plants treated with ABA + NaCl. However, the effect of Eth on salt-stressed plants was not significantly changed when Flu was applied to the plants (Figure 1A,B).

The levels of H_2_O_2_ and TBARS significantly increased, by 3.9- and 6.9-fold, respectively, in plants grown under salt stress compared with the control plants (Figure 1C,D). Compared with the control plants, the plants treated with Eth and ABA under unstressed conditions experienced less oxidative stress as measured by reduced levels of H_2_O_2_ and TBARS. These values decreased by 23.9% and 48.2% on Eth, and by 5.6% and 25.5% on ABA applications, respectively. The application of Eth and ABA to salt-stressed plants significantly reduced the increase in H_2_O_2_ (by 75.0% and 52.1%) and TBARS content (by 88.5% and 40.31%), respectively, compared with the plants subjected to NaCl treatment (Figure 1C,D). Further, application of ABA to salt-stressed plants treated with AVG resulted in an increased accumulation of H_2_O_2_ and TBARS content that was nearly equal to that of the NaCl treatment. However, the application of Flu to Eth-treated salt-stressed plants showed an increase in these parameters by 8.7% and 30.5% in comparison with Eth-treated salt-stressed plants (Figure 1C,D).

### 2.5. Effects of Eth, ABA, Flu, or AVG on ROS Accumulation in Mustard Grown under Salt Stress

**Figure 2:**
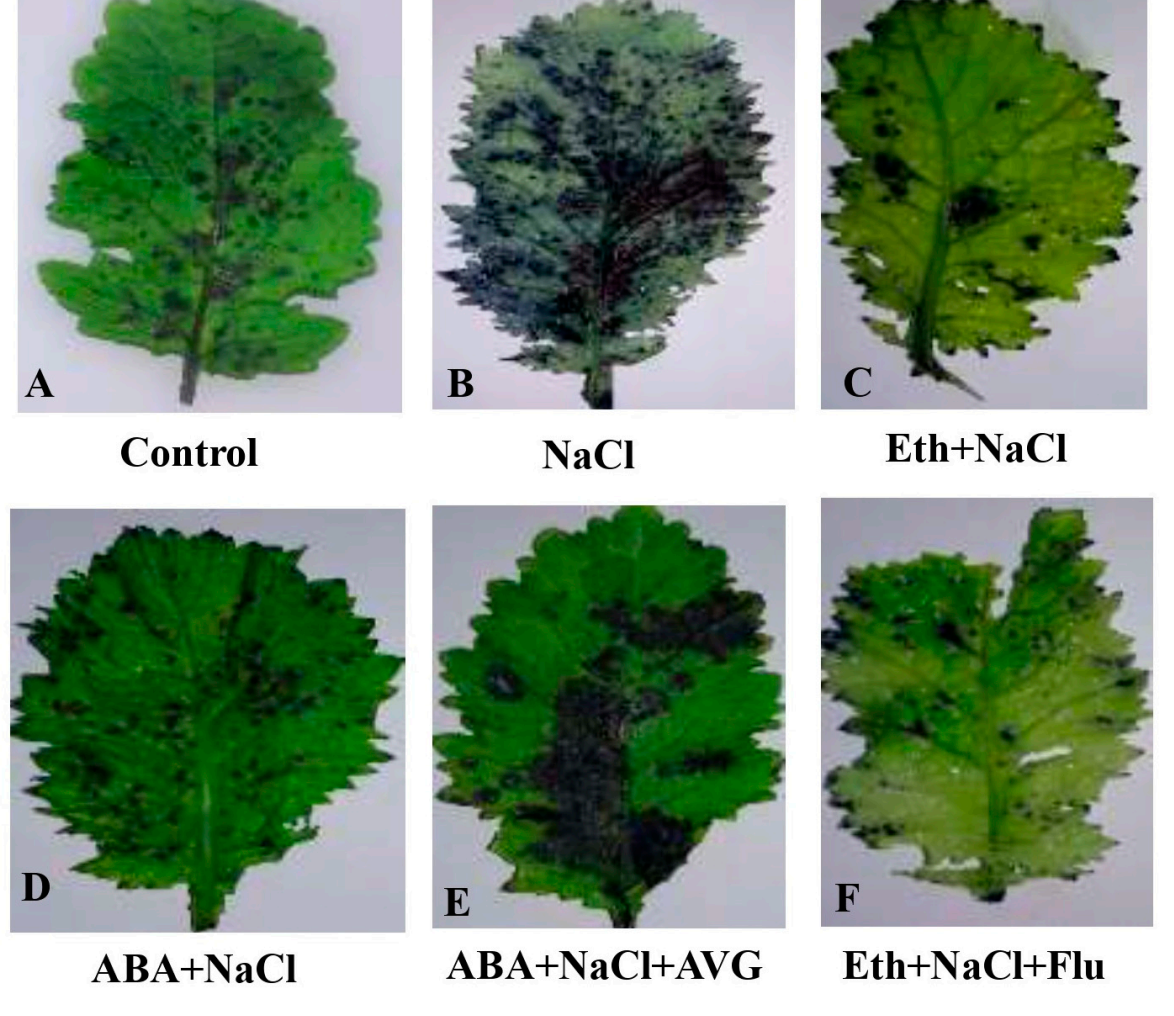
Visualization of superoxide ion by NBT staining in mustard (*Brassica juncea* L. cv. Pusa Vijay) seedlings grown under normal and 100 mmol NaCl stress conditions at 30 days after sowing, after foliar treatment of 2.0 mmol Eth, 25 µmol ABA, 25 mmol Flu, or 0.01 µmol AVG at 15 days after sowing. ABA: abscisic acid; AVG: aminoethoxyvinylglycine; Eth: ethephon; Flu: fluoridone.

As evident from Figure 2, the least accumulation was observed in the control plants (A). NaCl caused an increase in the presence of O_2_^−^ ions (B). However, when treated with either Eth (C) or ABA (D), the NaCl-exposed plants showed a few patches of NBT (nitro blue tetrazolium) blue staining when compared with NaCl treatment alone. Additionally, as shown by deep staining (E), the impact of Eth-induced alleviation of NaCl stress was entirely decreased when AVG was applied to plants that had been exposed to ABA-supplied NaCl stress. Flu-treated plants with NaCl treatment and Eth treatment displayed greater reductions in stained area and color intensity (F).

### 2.6. Effects of Eth, ABA, Flu, or AVG on S and N Assimilation and Antioxidant Enzyme Activity in Mustard Grown under Salt Stress

Salt-stress treatment decreased S content, but the content of cysteine and GSH significantly increased. The application of Eth and ABA separately showed improved S assimilation compared with the control (Table 4). The salt-stressed plants, when treated with Eth and ABA individually, improved S content (by 66.1% and 32.9%), cysteine (by 54.1% and 11.1%), and reduced glutathione (GSH) (by 9.7% and 3.6%), respectively, relative to the NaCl-treated plants. Additionally, AVG treatment applied to ABA-treated salt-stressed plants resulted in a drop in the above parameters by 17.2%, 16.8%, and 15.9%, respectively, compared with the ABA-treated salt-stressed plants. The salt-stressed plants treated with Eth and exposed to Flu showed a reduction in the content of S by 7.6% and cysteine by 2.6%; no change in GSH content was noticed compared with the ETH-treated salt-stressed plants (Table 4).

The content of N and the nitrate reductase (NR) activity in plants significantly decreased after exposure to NaCl. Eth and ABA supplementation boosted both N content and NR activity under unstressed conditions (Table 4). Also, individual Eth and ABA applications under salt-stressed conditions increased N content (by 84.3% and 45.2%) and NR activity (by 70.6% and 38.1%), respectively, compared with plants treated with NaCl (Table 4). Further, the application of AVG to ABA-treated salt-stressed plants resulted in a significant reduction in the N content and NR activity of the plants compared with ABA-applied salt-stressed plants. However, when salt-stressed plants treated with Eth were given Flu, no such reversal was seen, and the outcomes were noticeably comparable to those of the Eth-treated salt-stressed plants (Table 4).

Salt stress boosted the activity of APX and GR. These parameters greatly increased with the application of Eth alone, whereas the activity of these enzymes increased less with the application of ABA alone under unstressed conditions (Table 4). Under salt stress, individual treatment of Eth and ABA increased the activity of APX (by 83.0% and 30.2%) and GR (by 83.8% and 22.5%) relative to plants treated with NaCl. The activity of APX and GR was reduced by 47.0% and 39.4%, respectively, when AVG was applied in addition to the ABA treatment for salt-stressed plants. Flu, however, demonstrated reductions of only 10.0% and 5.2% in APX and GR activity, respectively, in contrast to the Eth-treated salt-stressed plants (Table 4).

### 2.7. Effect of Eth, ABA, Flu, or AVG on ACS Activity, ET Evolution, and ABA Content in Mustard Grown under Salt Stress

**Figure 3:**
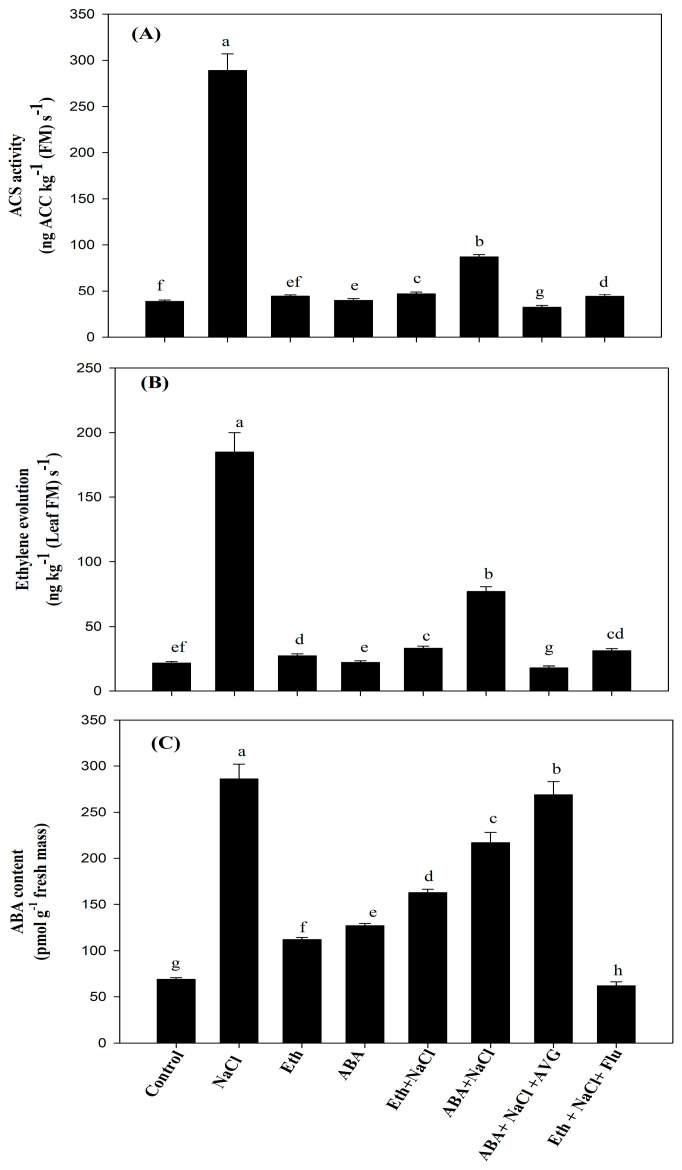
Activity of 1-aminocyclopropane-1-carboxylic acid synthase (ACS) (**A**), ET evolution (**B**), and ABA content (**C**) in mustard (*Brassica juncea* L. cv. Pusa Vijay) seedlings grown under normal and 100 mmol NaCl stress conditions at 30 days after sowing, after foliar treatment of 2.0 mmol Eth, 25 µmol ABA, 25 µmol Flu, or 0.01 µmol AVG at 15 days after sowing. Values are presented as means ± SE (n = 4). Data means denoted by the same letter are not significantly different at *p* ≤ 0.05 according to the LSD test. ABA: abscisic acid; AVG: aminoethoxyvinylglycine; Eth: ethephon; Flu: fluoridone.

ACS activity and ET evolution significantly increased under salt stress compared with control plants, increasing by 7.5- and 8.6-fold, respectively. However, the application of Eth and ABA separately under salt stress decreased ACS activity (by 83.7% and 69.8%) and ET evolution (by 82.2% and 58.3%) compared with salt-treated plants (Figure 3A,B). Furthermore, Eth application resulted in reduced ACS activity and ET evolution by 6.1% and 5.7%, respectively, in salt-stressed plants when Flu was administered to the plants, compared with plants treated with Eth + NaCl. In contrast, when salt-stressed plants treated with ABA were given AVG treatment, a significant decrease in ACS activity and ET evolution was noted. These parameters were decreased by 62.8% and 76.8%, respectively, compared with the salt-stressed plants treated with ABA (Figure 3A,B). ABA levels increased by 4.1 times in plants grown under salt stress. The increased ABA level was noted with Eth and ABA application under unstressed conditions, but the increase was less than with NaCl treatment. However, under salt-stressed conditions, the application of Eth and ABA reduced the ABA content (Figure 3C). Additionally, when AVG was applied to salt-stressed plants that received ABA treatment, the level of ABA increased significantly, by 23.9%, in comparison with plants treated with ABA and NaCl. However, when Flu was administered to plants that were exposed to salt stress after treatment with Eth, ABA content was reduced by 61.9% in comparison with plants treated with Eth and NaCl (Figure 3C).

### 2.8. Effect of Eth, ABA, Flu, or AVG on Stomatal Behavior in Mustard Grown under Salt Stress

**Figure 4:**
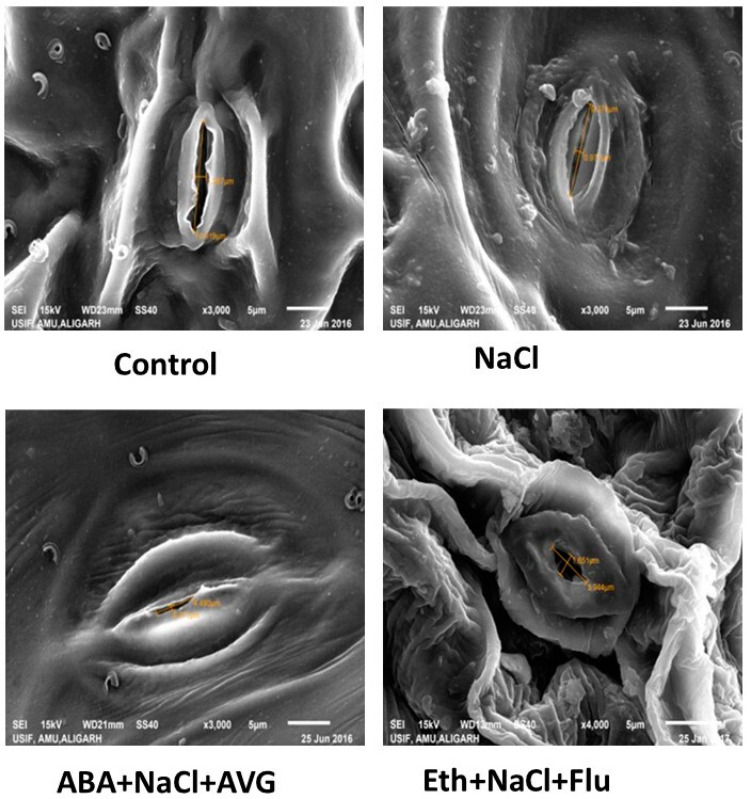
Stomatal behavior in mustard (*Brassica juncea* L. cv. Pusa Vijay) seedlings grown under normal and 100 mmol NaCl stress conditions at 30 days after sowing, after foliar treatment of 2.0 mmol Eth, 25 µmol ABA, 25 µmol Flu, or 0.01 µmol AVG at 15 days after sowing. ABA: abscisic acid; AVG: aminoethoxyvinylglycine; Eth: ethephon; Flu: fluoridone.

To better understand how guard cells respond to Eth and ABA treatment, electron microscopy was used. Compared with the control plants, the plants treated with NaCl had significantly smaller stomatal apertures. The stomatal opening (length and width) was 10.91 μm and 1.29 μm in control plants (Figure 3), but it was 9.37 μm and 0.97 μm in leaf samples from NaCl-treated plants. Continuing treatment with ABA + NaCl + AVG resulted in a decrease in stomatal aperture, with observed stomatal opening length and width measuring approximately 4.49 μm and 0.47 μm, respectively. Conversely, plants treated with Eth + NaCl + Flu did not exhibit as significant a reduction in stomatal aperture as that seen in plants treated with ABA + NaCl + AVG (Figure 4).

### 2.9. Principal Component Analysis (PCA)

**Figure 5:**
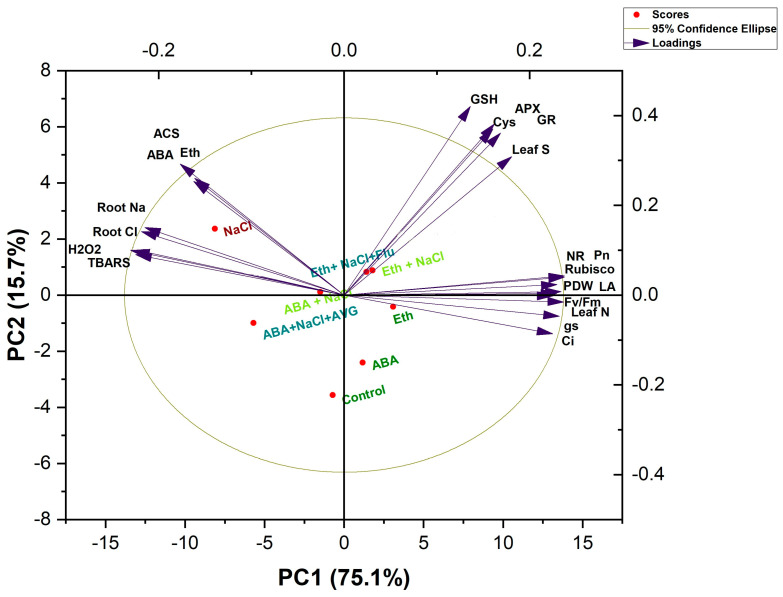
Principal component analysis (PCA) biplots to show connections between various treatments and variables in 30-days-old mustard (*Brassica juncea* L. cv. Pusa Vijay) seedlings. The diverse conditions are as follows: control; NaCl (100 mmol); Eth (2.0 mmol); ABA (25 µmol); AVG (0.01 µmol); Flu (25 µmol); Eth + NaCl; ABA + NaCl; ABA + NaCl + AVG; Eth + NaCl + Flu. ABA: abscisic acid; AVG: aminoethoxyvinylglycine (ethylene biosynthesis inhibitor); ET: ethylene; Flu: fluoridone (ABA biosynthesis inhibitor). The variables included ABA (abscisic acid), ACS (aminocyclopropane-1-carboxylic acid), APX (ascorbate peroxidase), Ci (intercellular CO_2_ concentration), Cys (cysteine), ET content, GR (glutathione reductase) activity, Gs (stomatal conductance), GSH (glutathione) content, H_2_O_2_ (hydrogen peroxide), K^+^ (potassium) content, LA (leaf area), N (nitrogen) content, Na^+^ (sodium) content, NR (nitrate reductase) activity, PDW (plant dry weight), Pn (net photosynthesis), PSII (maximum efficiency of PSII), Rubisco activity, S (sulfur) content, SOD (superoxide dismutase) activity, SPAD value, and TBARS (thiobarbituric acid reactive substances) content.

The PCA scores used to evaluate the effects of Eth and ABA on *B. juncea* plants under salt stress are shown in Figure 5. PC1 and PC2 explain 90.8% of the overall variation in the dataset. PC2 generated 15.7% of the variance, whereas PC1 provided 75.1% of it. The first two main components successfully disseminated all of the treatments (Figure 5). The observed parameters in the PCA biplot were classified into four components. The oxidative stress biomarkers (H_2_O_2_ and TBARS content) and root Na^+^ and Cl^−^ content values were all distributed along with the treatment of 100 mM NaCl. High salt stress influenced parameters such as H_2_O_2_ and TBARS content, ABA and Eth content, and ACS activity.

The parameters of growth, photosynthesis, and N metabolism were close to those of ethephon treatment without stress. On the other hand, in the presence of NaCl, antioxidants (APX, GR and GSH) and S and Cys content were close to the combined treatment of Eth + NaCl and Eth + NaCl + Flu compared with ABA +NaCl and ABA +NaCl +AVG (Figure 5). The parameters of plant growth and photosynthesis showed a negative correlation with oxidative stress biomarkers and Eth and ABA generation. The biplot makes it obvious that the antioxidants and S assimilation components were situated between oxidative stress and plant growth and photosynthesis, pointing to their potential role in mitigating salt stress. As a result, the biplot shows a relationship between Eth and ABA during the acclimation of *B. juncea* plants to salinity stress (Figure 5).

## 3. Discussion

Plants respond to salt stress through a complex network involving various physiological and biochemical systems, including hormone signals such as ET and ABA [38]. This study explores the crosstalk between ET and ABA under salt stress by conducting comprehensive biochemical and physiological analyses in mustard plants.

Screening data suggest that plant dry mass, leaf area, photosynthetic efficiency, ACS activity, and ET evolution significantly increased in all five cultivars of *Brassica juncea* under different doses of Eth (0, 0.5, 1.0, and 2.0 mmol). The cultivar Pusa Vijay showed much higher sensitivity to ET at all Eth levels, with the greatest response at 2.0 mmol, resulting in maximal growth and photosynthesis. The cultivar PM-28 showed the least or negligible sensitivity to ET (Table 1).

The process of seed germination is intricate, regulated by numerous hormones, and influenced by various environmental factors. Generally, ABA promotes seed dormancy and inhibits seed germination, while gibberellic acid and ET promote seed germination [39]. However, according to many studies [40,41], ET reverses the inhibitory effect of ABA on seed germination. Our findings find support from these studies (Table 2). ET facilitates seed germination by counteracting the effects of ABA [41,42,43]. In our study, it was observed that pretreatment of seeds with Eth accounted for the maximum germination percentage, germination index, and vigor index; however, ABA application showed a negative impact on the above attributes (Table 2). During seed germination, the application of ABA causes changes in the expression of ACC oxidase (ACO) rather than ACS, influencing ET biosynthesis [44]. ET also plays a crucial role in modulating sensitivity to ABA during the regulation of germination under salt stress [45]. It has been observed that ABA signaling primarily regulates the function of ETR1 and ETR2 in germinating seeds during salt stress [46]. ET also controls the expression of TaEXPA3, influencing germination by managing coleoptile ABA metabolism and ABA signaling through the expression of TaABI3 and TaABI5 [40]. Amylase plays an important role during seed germination by mobilizing starch, which acts as a source of energy for germinating seeds. According to the results, Eth was able to elicit the maximum levels of amylase, suggesting their role in seed germination (Table 2). Many studies have reported the potential involvement of Eth in inducing the activity of amylase [40,47]. Moreover, in germinating seeds, ET and ABA lower the ROS accumulation and elevate the level of antioxidants to mitigate the negative role of salt stress in seed germination [45]. These findings illustrate the antagonistic interaction between ET and ABA in regulating seed germination and starch metabolism, while they work synergistically in improving antioxidant defense machinery and seedling growth under salt stress.

The enhanced growth and photosynthesis in ET-sensitive Pusa Vijay might be due to its higher ability to inhibit receptors. It has been shown that the concentration of ET receptors and plant ET sensitivity are interdependent; a lower concentration of receptors shows more sensitivity of the plant to ET because a small amount of ET inactivates a higher percentage of the receptors [48,49]. The sensitivity of plants to ET, and its effects on stomatal movement and photosynthesis, are concentration-, species-, and growth-stage-specific [50,51,52,53,54]. Ethephon’s impact on photosynthetic efficiency varies, potentially increasing it through indirect effects on stomatal behavior and the direct modulation of Rubisco and carboxylation efficiency [54]. It has been shown that the key enzyme of the ET biosynthetic pathway, ACS, shows a positive correlation with the photosynthetic efficiency of mustard cultivars showing a degree of variability in photosynthetic capacity [50]. Enhanced stomatal conductance, intercellular CO₂, and net photosynthesis were particularly notable in the ET-sensitive cultivars like Pusa Vijay; however, the ET-insensitive cultivars showed no significant changes in photosynthetic efficiency with the application of Eth [55,56]. Eth also increased the leaf area of the mustard, supporting earlier findings that link Eth application to enhanced leaf area in mustard through increased ET evolution [50,56]. ET regulates stress responses and plant development, with dose-dependent effects that vary from growth inhibition at high ET concentrations in *Triticum aestivum* and *Cucumis sativus* to the activation of defense signaling at low concentrations [20,57].

The individual application of Eth or ABA mitigated the salt stress, but Eth demonstrated more pronounced results compared with ABA. As expected, higher concentrations of Na^+^ and Cl^−^ ions were observed in the salt-stressed plants, which can disrupt amino acid biosynthesis, protein synthesis, mRNA processing, transcription, cellular homeostasis, and nutrient and lipid metabolism [58,59,60]. The salt tolerance of different plant species has been reported to be improved by both the exogenous application of ABA [21,61,62,63] and Eth [17,20] and by limiting Na^+^ and Cl^−^ ions. In this study, the negative influence of salt stress was attributed to a more significant accumulation and translocation of Na^+^ and Cl^−^ into leaves, leading to higher H_2_O_2_ and TBARS content in the leaves, causing oxidative bursts that negatively impacted the growth and photosynthetic performance of mustard. However, Eth and ABA application reduces oxidative stress by enhancing the antioxidant system, as reported in early studies [5,21], but out of the two, Eth showed a more pronounced effect.

Plant growth and photosynthesis are negatively impacted by salt stress due to changes in the chloroplast ultrastructure, which include modifications to the thylakoid membrane, chloroplast protein complexes, photosystem II, and Rubisco activity [30]. It was discovered that ET may shield PSII activity and photosynthesis from stress by minimizing chlorophyll loss [5,64]. Similarly, under Cd stress, various Eth treatments enhanced the Fv/Fm, Rubisco, and photosynthesis of mustard [65]. Although at high concentrations, Eth damages the photosynthetic mechanism in response to different stresses its effect on photosynthetic protection was proved to be more concentration-dependent [5,66]. According to this study, plants subjected to 100 mM NaCl produced an excessive amount of ET, which decreased their ability to photosynthesize. This could be due to the formation of stress ET that caused photosynthetic repression [5,17]. Among the individual applications of Eth and ABA, the Eth application maximally decreased stress ET and brought it to the ideal level required by the plant for positively impacting photosynthesis. The possible reason may be the optimal ET-induced maximum enhancement in antioxidant potential that reduced oxidative stress. Abiotic stress factors are primarily regulated by enzymatic and non-enzymatic antioxidants. Our previous reports have shown that Eth treatment promotes the activity of SOD, APX, and GR by optimizing endogenous ET [5,64,65,66]. Further, GR controls GSH biosynthesis for the optimal S metabolism [67]. In this study, higher GR activity through Eth supplementation could be attributed to induced salt tolerance by the regulation of cysteine and GSH biosynthesis. The link between ET, GSH, and S for cadmium tolerance in mustard and wheat has already been demonstrated [65,67]. Moreover, increased GSH synthesis may also be responsible for the decrease in stress ET formation that not only helped with the reduction in excess endogenous ET but also acted as a potential antioxidant [5]. Lower or optimal ET evolution and increased photosynthetic efficiency were the results of this antioxidant activity. Further, the accessible ABA aids in the synthesis of more GSH for efficient ROS detoxification under stressful conditions [68]. Here, the application of ABA under salt stress led to an increase in APX and GR activity to counteract the damage to photosynthetic machinery caused by ROS. However, the individual application of Eth was found to be more effective than ABA in maintaining growth and photosynthesis of mustard plants. This is probably due to a more controlled impact on N and S assimilation by ET than by ABA alone.

In Arabidopsis plants, it was observed that both ET and ABA application resulted in half-open stomata [26,31]. In our results, the application of inhibitors led to a reduction in stomatal opening, with the most significant decrease observed in the treatment where ET was eliminated. Reduced H_2_O_2_ levels and increased antioxidant activity are the results of both Eth and ABA treatment, which also leads to reduced stomatal closure [38]. However, to clarify if it is governed by an ET–ABA connection or if they function in parallel, more investigation is required. It can be said that, depending on the surrounding circumstances, ET and ABA might affect each other’s biosynthetic or signaling pathways, which in turn control stomatal mobility [69,70,71,72].

Earlier, ET–nitrogen synergism was shown to induce tolerance to copper stress by modulating the antioxidant system and N metabolism, resulting in improved photosynthetic capacity of mustard plants [9]. This study convincingly illustrates that the suppressed photosynthetic function under salt stress can be ameliorated through the separate application of Eth and ABA, which effectively mitigated salt-induced stress in mustard. This mitigation was achieved by enhancing the antioxidant capacity and stimulating S and N metabolism. Notably, ET exhibited a more significant impact compared with ABA. However, here the effect of ABA on photosynthesis was found to be dependent on ET, since inhibiting ET biosynthesis with AVG inhibited the observed increase in photosynthesis by ABA. The decreased antioxidant activities and altered S and N metabolism could be the cause of this reduction. Cao et al. [73] suggested a link between ABA biosynthesis and S metabolism in Arabidopsis plants by showing how sulfate availability affects endogenous ABA accumulation. Further, in mustard plants, ABA coordination with N was found to alleviate salinity-inhibited photosynthetic potential by improving proline accumulation and antioxidant activity [21]. ABA indirectly, via different signals, controls S and N metabolism. ET biosynthesis and signaling, however, is more related and is found to govern these pathways more efficiently [30,65,74]. Our study clearly indicates why ET signaling is more crucial: through the higher induction of S, Cys, and GSH and by influencing the higher N assimilation (N content and NR activity) capacity of mustard. Overall, the increase in photosynthesis with both Eth and ABA improved the growth of plants, as evidenced by increased leaf area and plant dry mass, but the more pronounced effect was that of Eth. The results are in alignment with the earlier studies [21,30]. This indicates that ET signaling is necessary in combination with ABA to enhance growth and photosynthesis under salt stress, whereas inhibiting ABA has little effect on ET action.

## 4. Materials and Methods

### 4.1. Plant Material and Growth Conditions

Seeds of five different cultivars (Pusa Vijay, Pusa Jagannath, Pusa Agrani, PM-27, and PM-28) of mustard (*Brassica juncea* L. Czern and Coss.) were surface sterilized with 0.01% HgCl_2_ solution and were repeatedly washed with double-distilled water before sowing in 23 cm diameter earthen pots filled with 5 kg of reconstituted soil (sand:clay:peat 70:20:10 by dry weight). The pots were kept in a naturally illuminated greenhouse of the Department of Botany, Aligarh Muslim University Aligarh, India, with photosynthetically active radiation (PAR) ∼630 µmol m^−2^ s^−1^, average day/night temperatures of 22/14 ± 3 °C, and relative humidity of 62 ± 5%. At the 10-day seedling stage, all five mustards were subjected to the foliar treatment of 0, 0.5, 1.0, or 2.0 mmol Eth to screen out the most responsive dose of Eth. Measurements were taken at 30 days after sowing (DAS). The most effective dose of 2.0 mmol Eth and the cultivar Pusa Vijay that responded maximally were chosen for the next experiment. The number of replicates (n) used for each treatment was four (n = 4).

In the next experiment, the sterilized seeds were randomly placed on wet filter paper in Petri plates for germination at 28 ± 2 °C. The seeds were pretreated with 2.0 mmol Eth and 25 µmol ABA (Sigma) with or without salt stress. Salt-stressed seeds were subjected to 100 mmol NaCl for three days. Measurements of germination-related attributes were taken from 5-day-old seedlings. ABA was applied exogenously after dissolving in potassium hydroxide solution. The concentration of ABA was chosen from our previously published study [21]. To establish their respective roles, biosynthesis inhibitors of Eth and ABA were also used: AVG as Eth biosynthesis inhibitor and Flu as ABA biosynthesis inhibitor. A concentration of 0.1 µmol of AVG and 25 µmol of Flu was given in the experiment. The concentration of inhibitors was based on the study of Mir et al. [75] and Wu et al. [76]. Each Petri plate contained 50 seeds (n = 50), the number of replicates for each treatment was 4 (n = 4), and the germination percentage was determined by assessing the standard radicle emergence of the seeds.

Later in the experiment, the same set of treatments was used. In the next experiment, plants were subjected to 100 mmol NaCl concentration at 10 DAS. Foliar application of ethephon and ABA on stressed and unstressed plants was performed at 15 DAS with 0.5% surfactant Teepol. Moreover, simultaneously, their inhibitors, 0.01 µmol AVG and 25 µmol Flu, were given at 15 DAS with 0.5% surfactant Teepol. At 30 DAS, measurements were taken. Four replicates for each treatment were taken (n = 4).

### 4.2. Seed Germination Parameters

The standard radicle emergence of each seed in a Petri plate was used to calculate the germination percentage. The germination index (GI) was calculated by using the formula, ∑(Gt/Tt); where Gt denotes number of germinating seeds in ‘t’ days, and Tt represents the days corresponding to Gt. Vigor index (VI) of seeds was calculated by the formula, VI = GI × SDW/MGT; where SDW is seed dry weight and MGT is mean germination time, which was calculated as MGT = ∑Ti × ∑Ni/∑Ni. Ni stands for number of new germinating seeds at time ‘Ti’.

### 4.3. Determination of Amylase Activity

Amylase activity was assessed using the starch–iodine protocol outlined by Collins et al. [77]. After germination, 0.5 g of seeds were collected and homogenized in a pre-chilled mortar and pestle with 6 mL of 50 mM Tris-HCl (pH 7.5) containing 1% PVP and 15 mM 2-mercaptoethanol. The homogenate was then centrifuged at 10,000× *g* for 30 min at 4 °C. The resulting supernatant was used to determine enzyme activity. Enzyme units were determined based on the amount of enzyme required to achieve a 50% reduction in the original color intensity.

### 4.4. Photosynthetic Traits

Net photosynthetic rate, stomatal conductance, and intercellular CO_2_ concentration were measured with the help of photosynthesis system (CI-340; CID, Biosciences, Camas, WA, USA) on fully expanded young leaves. The chlorophyll fluorescence was measured with the help of chlorophyll fluorometer Model JUNIOR-PAM (Heinz Walz, Germany).

Rubisco activity was determined spectrophotometrically by monitoring NADH oxidation at 30 °C at 340 nm [78]. Leaf tissue (1.0 g) was homogenized using a chilled mortar and pestle with ice-cold extraction buffer containing 0.25 M Tris-HCl (pH 7.8), 0.05 M MgCl_2_, 0.0025 M EDTA, and 37.5 mg DTT. The homogenate was centrifuged at 4 °C at 10,000× *g* for 10 min. The resulting supernatant was used to assay the enzyme. The reaction mixture contained 100 mM Tris-HCl (pH 8.0), 40 mM NaHCO_3_, 10 mM MgCl_2_, 0.2 mM NADH, 4 mM ATP, 0.2 mM EDTA, 5 mM DTT, and 1 U of 3-phosphoglycerate kinase. The activity was estimated after the addition of enzyme extract and 0.2 mM ribulose-1, 5-bisphosphate (RuBP).

### 4.5. Determination of Growth Parameters

The plants were dried in a hot-air oven at 80 °C until constant mass. The dried material was weighed on an electrical balance, and the weight was recorded as a whole plant dry mass. Leaf area was measured with a leaf-area meter (LA 211, Systronics, New Delhi, India).

### 4.6. Determination of Sulfur Content

Total S in plant samples was estimated according to the turbidimetric method of Chesnin and Yien [79]. Details are given in Supplementary File S1.

### 4.7. Determination of Cysteine Content

The content of cysteine in leaves was determined spectrophotometrically, adopting the method of Giatonde [80]. Details of the protocol are given in Supplementary File S1.

### 4.8. Determination of Glutathione Content

Glutathione content was determined spectrophotometrically, following the method of Anderson [81]. Fresh leaf tissue (0.5 g) was crushed in 2.0 mL of 5% sulfosalicylic acid using a pre-chilled mortar and pestle at 4 °C. The homogenized material was then centrifuged at 10,000× *g* for 10 min. To 0.5 mL of the resulting supernatant, 0.6 mL of phosphate buffer (100 mM, pH 7.0) and 40 mL of 5,5′-dithiobis-2-nitrobenzoic acid were added. After 2 min, the absorbance was measured at 412 nm.

### 4.9. Determination of Nitrogen Content

Leaf N content was estimated using the Kjeldahl digestion method as described by Lindner [82]. Specifics of the procedure are given in Supplementary File S1.

### 4.10. Determination of Nitrate Reductase Activity

The activity of nitrate reductase (EC 1.6.6.1) in leaves was measured by preparing an enzyme extract using the method of Kuo et al. [83]. Details of the procedure are given in Supplementary File S1.

### 4.11. Assay of Antioxidant Enzymes

The activity of GR was determined by adopting the method of Foyer and Halliwell [84] and by monitoring the GSH-dependent oxidation of NADPH at 340 nm. The activity of APX was assayed by the method of Nakano and Asada [85] with slight modifications. Fresh leaves and germinating seeds of the second experiment were homogenized with mortar and pestle homogenizers with an extraction buffer containing 0.05% (*v*/*v*) Triton X-100 and 1% (*w*/*v*) polyvinylpyrrolidone in 100 mM potassium phosphate buffer (pH 7.0). The homogenate was centrifuged at 15,000× *g* for 20 min. The resulting supernatant obtained by centrifugation was used to assay the enzyme GR (EC; 1.6.4.2). For the assay of APX, extraction buffer was added with 2 mM AsA. The details of the procedure of both the APX and GR assays have been described earlier in Mir et al. [86].

### 4.12. Ion Accumulation

#### Digestion of Plant Tissues

Oven-dried plant tissues were taken in 50 mL volumetric flask. In this flask, 2 ml concentrated HNO_3_ was added, and the mixture was heated on a hot plate until a brown effervescence was observed. On stopping of the effervescence, TAM (tri-acid mixture; nitric acid + sulfuric acid + perchloric acid in the ratio of 10:5:4) solution was added until a clear solution was obtained. The entire mixture was left to dry on the hot plate. Once dried, 50 mL of DDW was added, shaken, and then transferred into another 50 mL volumetric flask. The final volume was made up to the mark by adding DDW. Na^+^ in digested root samples were determined using a flame photometer (Systronics). To estimate the Cl^−^ content in the digested root samples, 50 mL of digested plant samples was placed in a flask, and 2 ml of 5% K_2_CrO_4_ indicator solution was added. The solution was then titrated against a 0.02N silver nitrate solution, and the Cl^−^ was calculated using the following formula:

$$Cl^{-}\;(\mathrm{mg}\;L^{-1})\;=\;\frac{(A-B)\;N\;\mathrm{of}\;{\mathrm{AgNO}}_{3}\;\times \;1000\;\times \;35.5}{\mathrm{mL}\;\mathrm{sample}}$$

where A is ml titration for the sample and B is ml titration for the blank.

The digested plant samples were analyzed for Na^+^ and Cl^−^ content using tri-acid mixture (TAM), comprising nitric acid, sulfuric acid, and perchloric acid in a ratio of 10:5:4. The Na^+^ content was determined using a flame photometer (Khera-391: Khera Instruments, New Delhi, India), while the Cl^–^ content was assessed through titration against a 0.02 N silver nitrate solution with 5% potassium chromate (K_2_CrO_4_) serving as the indicator.

### 4.13. Determination of TBARS Content

Contents of TBARS were measured according to Dhindsa et al. [87] by recording absorbance at 532 nm and corrected for non-specific turbidity by subtracting the absorbance at 600 nm. The TBARS content was calculated using its extinction coefficient of 155 mM^−1^ cm^−1^.

### 4.14. Determination of H_2_O_2_ Content

The assay of H_2_O_2_ was made following Okuda et al. [88]. Leaves (0.5 g) were ground in ice-cold 200 mM perchloric acid. After centrifugation at 1200× *g* for 10 min, perchloric acid of the supernatant was neutralized with 4M KOH. The insoluble potassium perchlorate was eliminated by centrifugation at 500× *g* for 3 min. The reaction was started by the addition of peroxidase, and an increase in the absorbance was recorded at A_590_ for 3 min.

### 4.15. Measurement of ACS Activity, Ethylene Evolution, and ABA Content

The activity of ACS (EC 4.4.1.14) was measured by adopting the method of Avni et al. [89]. Leaf tissue weighing 5.0 g was grounded in 100 mM HEPES buffer (pH 8.0) supplemented with 4 mM dithiothreitol (DTT), 2.5 mM pyridoxal phosphate, and 25% polyvinylpyrrolidone (PVP). Following homogenization, the mixture was centrifuged at 12,000× *g* for 15 min. Subsequently, 1 mL of the supernatant was transferred to a 30-mL tube, to which 0.1 mL of 5 mM AdoMet was added, and the mixture was incubated for 2 h at 22 °C. The formation of ACC was assessed by its conversion to ethylene through the addition of 0.1 mL of 20 mM HgCl_2_ followed by 0.1 mL of a 1:1 mixture of saturated NaOH/NaCl. The mixture was then placed on ice for 10 min. For the control set, AdoMet was omitted.

Ethylene was identified based on the retention time and quantified by comparison with peaks from standard ethylene. The details of the protocol are reported by Fatma et al. [30]. Specifics of the protocol are given in Supplementary File S1.

The ABA levels were assessed following the method outlined by Hung and Kao [90] with minor adjustments. An ABA immunoassay detection kit (PGR-1; Sigma–Aldrich, St. Louis, MO, USA) was utilized according to the manufacturer’s instructions to determine ABA content. Further procedural information can be found in Fatma et al. [30]. Details are provided in Supplementary File S1.

### 4.16. Physiological Measurements of Guard Cells

Upper leaves of 30-day-old plants grown under natural day/night conditions with photosynthetically active radiation (PAR) ∼630 µmol m^−2^ s^−1^ and average day/night temperatures of 22/14 ± 3 °C were plugged from each with different treatments and were fixed in 2.5% glutaraldehyde. The samples were collected in the morning around 10:00 A.M. Stomatal pictures were taken using scanning electron microscopy (JSM-6510 LV, JEOL, Tokyo, Japan; available in the USIF: University Sophisticated Instruments Facility of the University, AMU, Aligarh, India). The details are provided in our earlier work by Mir et al. [91].

### 4.17. Histochemical Detection of ROS

The histochemical staining method using NBT was utilized for the assay of the accumulation of O_2_^−^ in leaves, following the procedure outlined by Kumar et al. [92] with minor adjustments. Samples from each treatment were submerged in a 1 mg/mL NBT solution prepared in 10 mM phosphate buffer (pH 7.8) and kept at room temperature under light conditions for 6 h. This led to the appearance of blue spots indicating NBT staining. To enhance clarity, the stained samples were subjected to boiling in ethanol for discoloration.

### 4.18. Statistical Analysis

Data were analyzed statistically with analysis of variance (ANOVA) using SPSS v18.0 for Windows (IBM Corporation, New York, NY, USA). Principal component analysis between different variables was performed using OriginPro (v 9.8) for Windows. The Least significant difference (LSD) was calculated for mean separation for significant differences among treatments at *p* < 0.05 levels.

## 5. Conclusions

Ethylene enhances salt tolerance independently and through interaction with ABA, promoting S and N assimilation via increased APX, GR, and GSH, leading to enhanced growth and photosynthesis. Ethylene’s effects surpassed those of ABA, which showed variable responses dependent on ethylene levels. The suppression of Eth decreased ABA-induced Eth generation and antioxidant enzyme activity. Ethylene pretreatment improved seed germination under salt stress, contrasting with ABA’s germination inhibition reversal. The complex interplay between Eth and ABA highlights their regulatory roles in salt-stress responses, emphasizing the need for further research into their interactive and feedback mechanisms.

## Figures and Tables

**Table 1:** Plant dry mass, leaf area, net photosynthesis, stomatal conductance, intercellular CO_2_ concentration, ACS activity, and ET evolution in five cultivars of mustard (*Brassica juncea* L.) at 30 days after sowing following foliar Eth treatment of 10-days-old seedlings. Values are means ± SE (n = 4). Data means followed by the same letter are not significantly different at *p* ≤ 0.05 according to the LSD test. ACS: 1-1minocyclopropane carboxylic acid (ACC) synthase; ET: ethylene; Eth: ethephon.

Cultivar	Ethephon (mmol)	Plant Dry Mass (g plant^−1^)	Leaf Area (cm^2^ plant^−1^)	Net Photosynthesis (µmol CO_2_ m^−2^ s^−1^)	Stomatal Conductance (mmol CO_2_ m^−2^ s^−1^)	Intercellular CO_2_ Concentration (µmol CO_2_ mol^−1^)	ACS Activity (ng ACC kg^−1^ (FM) s^−1^)	Ethylene (ng kg^−1^ (FM) s^−1^)
PM-27	0 (control)	1.14 ± 0.10 ^o^	100.5 ± 7.1 ^n^	9.7 ± 1.0 ^j^	268.1 ± 11.5 ^m^	221.9 ± 25.4 ^m^	32.1 ± 2.0 ^h^	19.6 ± 1.5 ^g^
	0.5	1.38 ± 0.15 ^l^	108.3 ± 8.1 ^j^	10.1 ± 1.4 ^j^	286.6 ± 12.6 ^l^	239.5 ± 27.5 ^ij^	33.4 ± 2.4 ^g^	20.8 ± 1.8 ^ef^
	1.0	1.57 ± 0.19 ^jk^	115.5 ± 9.1 ^gh^	11.6 ± 1.8 ^hi^	317.1 ± 13.3 ^i^	267.5 ± 29.8 ^g^	35.4 ± 3.3 ^f^	22.7 ± 1.9 ^de^
	2.0	1.68 ± 0.1 ^j^	121.5 ± 8.3 ^ef^	13.8 ± 1.6 ^f^	359.6 ± 12.3 ^g^	295.8 ± 28.3 ^d^	38.6 ± 3.2 ^cd^	25.1 ± 2.6 ^c^
PM-28	0 (control)	1.11 ± 0.10 ^o^	100.7 ± 6.4 ^n^	8.3 ± 1.0 ^k^	255.2 ± 10.4 ^p^	219.6 ± 23.4 ^m^	31.3 ± 1.8 ^h^	18.5 ± 1.6 ^gh^
	0.5	1.32 ± 0.11 ^m^	107.3 ± 6.8 ^j^	8.6 ± 1.3 ^jk^	271.4 ± 11.6 ^m^	234.5 ± 24.6 ^jk^	32.6 ± 1.9 ^gh^	19.4 ± 1.8 ^g^
	1.0	1.39 ± 0.15 ^l^	112.3 ± 7.6 ^i^	9.6 ± 1.7 ^j^	283.3 ± 12.6 ^l^	256.6 ± 25.^gh^	33.6 ± 2.4 ^fg^	20.3 ± 1.9 ^f^
	2.0	1.48 ± 0.14 ^k^	117.1 ± 7.3 ^g^	11.4 ± 1.4 ^i^	324.6 ± 12.7 ^h^	285.9 ± 25.6 ^ef^	36.7 ± 2.1 ^de^	22.5 ± 2.1 ^de^
Pusa Agrani	0 (control)	1.23 ± 0.10 ^n^	102.6 ± 5.9 ^lm^	11.4 ± 1.0 ^i^	298.3 ± 11.7 ^k^	224.4 ± 25.l^m^	33.2 ± 2.2 ^g^	21.5 ± 1.4 ^ef^
	0.5	1.51 ± 0.07 ^k^	113.3 ± 4.6 ^hi^	12..1 ± 1.4 ^h^	329.4 ± 12.8 ^h^	244.8 ± 27.8 ^i^	35.1 ± 3.1 ^f^	23.1 ± 1.8 ^d^
	1.0	1.78 ± 0.17 ^hi^	124.2 ± 9.3 ^de^	13.8 ± 1.8 ^f^	360.3 ± 13.8 ^fg^	278.8 ± 30.2 ^f^	36.7 ± 3.6 ^ef^	25.3 ± 2.0 ^c^
	2.0	1.92 ± 0.14 ^g^	130.3 ± 8.1 ^c^	16.9 ± 1.7 ^c^	419.7 ± 10.6 ^c^	315.7 ± 29.6 ^c^	40.5 ± 3.3 ^c^	28.6 ± 2.6 ^b^
Pusa Jagannath	0 (control)	1.73 ± 0.10 ^j^	103.7 ± 7.3 ^l^	12.1 ± 0.96 ^h^	311.8 ± 11.9 ^j^	224.6 ± 29.5 ^l^	35.1 ± 2.4 ^f^	23.4 ± 1.5 ^d^
	0.5	2.13 ± 0.07 ^f^	117.8 ± 8.7 ^g^	13.1 ± 11.6 ^g^	356.6 ± 12.5 ^g^	249.5 ± 31.3 ^hi^	37.5 ± 3.3 ^de^	25.3 ± 2.0 ^c^
	1.0	2.54 ± 0.07 ^d^	128.4 ± 12.7 ^d^	14.9 ± 1.9 ^e^	386.2 ± 15.1 ^de^	289.7 ± 33.7 ^e^	39.2 ± 3.8 ^cd^	27.6 ± 2.6 ^bc^
	2.0	2.75 ± 0.47 ^c^	134.9 ± 12.5 ^b^	18.6 ± 1.7 ^ab^	457.6 ± 13.3 ^b^	345.4 ± 33.4 ^b^	43.5 ± 3.6 ^ab^	31.4 ± 2.7 ^a^
Pusa Vijay	0 (control)	1.82 ± 0.09 ^h^	106.2 ± 7.6 ^k^	12.2 ± 1.3 ^h^	312.8 ± 12.6 ^j^	226.1 ± 31.1 ^l^	35.7 ± 3.0 ^ef^	23.4 ± 1.5 ^d^
	0.5	2.27 ± 0.26 ^e^	123.5 ± 11.5 ^e^	13.5 ± 1.7 ^f^	365.6 ± 13.1 ^f^	255.9 ± 33.7 ^h^	38.6 ± 3.7 ^cd^	25.6 ± 2.1 ^c^
	1.0	3.09 ± 0.34 ^b^	135.4 ± 15.6 ^b^	15.6 ± 1.9 ^d^	396.7 ± 17.9 ^d^	299.1 ± 36.4 ^d^	40.3 ± 4.1 ^c^	28.5 ± 2.6 ^b^
	2.0	3.26 ± 0.31 ^a^	141.5 ± 14.2 ^a^	19.4 ± 1.56 ^a^	469.6 ± 14.6 ^a^	358.3 ± 35.8 ^a^	45.3 ± 4.0 ^a^	32.3 ± 2.8 ^a^

**Table 2:** Percent germination, germination index, vigor index, amylase activity, H_2_O_2_ content, APX activity, and GR activity in 5-days-old mustard (*Brassica juncea* L. cv. Pusa Vijay) seedlings grown under normal or 100 mmol NaCl stress conditions after seeds were pretreated with 2.0 mmol Eth, 25 µmol ABA, 25 µmol Flu, or 0.1 µmol AVG. Values are presented as means ± SE (n = 4). Data means followed by the same letter are not significantly different at *p* ≤ 0.05 according to the LSD test. ABA: abscisic acid; APX: ascorbate peroxidase; AVG: aminoethoxyvinylglycine; Eth: ethephon; GR: glutathione reductase; Flu: fluoridone.

Treatment	Germination Percentage (%)	Germination Index (%)	Vigor Index (%)	Amylase Activity (U g^−1^ Protein min^−1^)	H_2_O_2_ Content (nmol g^−1^ FW)	APX Activity (U g^−1^ Protein min^−1^)	GR Activity (U g^−1^ Protein min^−1^)
Control	72 ± 1.9 ^b^	21.2 ± 0.67 ^b^	3.2 ± 0.10 ^b^	82.5 ± 2.4 ^b^	46.3 ± 1.2 ^f^	0.68 ± 0.02 ^g^	0.27 ± 0.01 ^f^
NaCl	46 ± 1.2 ^e^	10.5 ± 0.299 ^e^	0.9 ± 0.02 ^e^	44.7 ± 1.2 ^e^	198.4 ± 5.4 ^a^	1.07 ± 0.03 ^e^	0.33 ± 0.02 ^e^
Eth	82 ± 2.4 ^a^	24.8 ± 0.72 ^a^	3.6 ± 0.11 ^a^	105.6 ± 3.3 ^a^	31.5 ± 0.8 ^h^	2.25 ± 0.06 ^c^	0.46 ± 0.03 ^c^
ABA	21 ± 0.4 ^f^	6.3 ± 0.72 ^f^	0.7 ± 0.02 ^f^	39.4 ± 1.0 ^f^	38.2 ± 0.9 ^g^	2.06 ± 0.06 ^d^	0.36 ± 0.01 ^de^
Eth + NaCl	58 ± 1.5 ^d^	14.2 ± 0.68 ^d^	1.1 ± 0.04 ^d^	66.8 ± 2.1 ^d^	53.7 ± 1.4 ^e^	3.41 ± 0.08 ^a^	0.67 ± 0.03 ^a^
ABA + NaCl	13 ± 0.1 ^g^	1.7 ± 0.04 ^g^	0.5 ± 0.01 ^g^	38.9 ± 0.6 ^fg^	71.8 ± 1.9 ^c^	2.21 ± 0.06 ^cd^	0.53 ± 0.01 ^bn^
ABA + NaCl +AVG	7 ± 0.3 ^h^	1.3 ± 0.05 ^h^	0.3 ± 0.01 ^h^	23.2 ± 1.0 ^h^	81.2 ± 1.9 ^b^	0.84 ± 0.02 ^f^	0.29 ± 0.01 ^fg^
Eth + NaCl + Flu	60 ± 1.4 ^c^	15.2 ± 0.39 ^c^	1.5 ± 0.03 ^c^	76.5 ± 1.8 ^c^	62.3 ± 1.4 ^d^	2.82 ± 0.09 ^b^	0.56 ± 0.01 ^b^

**Table 3:** Plant dry mass, leaf area, net photosynthesis, stomatal conductance, intercellular CO_2_ concentration, chlorophyll fluorescence (Fv/Fm), and Rubisco activity of mustard (*Brassica juncea* L. cv. Pusa Vijay) grown under normal and 100 mmol NaCl stress conditions at 30 days after sowing, and after foliar treatment of 2.0 mmol Eth, 25 µmol ABA, 25 µmol Flu, or 0.01 µmol AVG at 15 days after sowing. Values are presented as means ± SE (n = 4). Data means followed by the same letter are not significantly different at *p* ≤ 0.05 according to the LSD test. ABA: abscisic acid; AVG: aminoethoxyvinylglycine; Eth: ethephon; Flu: fluoridone.

Treatment	Plant Dry Mass (g plant^−1^)	Leaf Area (cm^2^ Plant^−1^)	Net Photosynthesis (µmol CO_2_ m^−2^ s^−1^)	Stomatal Conductance (mmol CO_2_ m^−2^ s^−1^)	Intercellular CO_2_ Concentration (µmol CO_2_ mol^−1^)	Fv/Fm	Rubisco Activity (µmol CO_2_ mg^−1^ Protein min^−1^)
Control	2.42 ± 0.23 ^f^	102.1 ± 5.76 ^e^	14.1 ± 1.12 ^e^	306 ± 23.7 ^f^	221 ± 23.1 ^f^	0.72 ± 0.04 ^f^	0.84 ± 0.069 ^f^
NaCl	1.32 ± 0.12 ^h^	49.2 ± 2.98 ^g^	5.8 ± 0.63 ^g^	214 ± 13.9 ^h^	131 ± 14.2 ^h^	0.42 ± 0.02 ^h^	0.32 ± 0.018 ^h^
Eth	3.54 ± 0.3 ^a^	138.1 ± 8.42 ^a^	19.5 ± 1.65 ^a^	456 ± 27.4 ^a^	348 ± 24.7 ^a^	0.93 ± 0.05 ^a^	1.39 ± 0.11 ^a^
ABA	3.19 ± 0.30 ^b^	127.3 ± 7.81 ^b^	18.3 ± 1.47 ^b^	410 ± 29.6 ^b^	302 ± 26.1 ^b^	0.87 ± 0.05 ^b^	1.24 ± 0.079 ^b^
Eth + NaCl	2.94 ± 0.28 ^c^	122.4 ± 7.77 ^b^	18.1 ± 1.55 ^b^	386 ± 26.6 ^c^	247 ± 23.5 ^c^	0.85 ± 0.05 ^c^	1.21 ± 0.93 ^c^
ABA + NaCl	2.79 ± 0.27 ^d^	113.3 ± 7.14 ^c^	16.5 ± 1.47 ^c^	361 ± 25.8 ^d^	226 ± 24.3 ^d^	0.74 ± 0.04 ^d^	1.14 ± 0.057 ^d^
ABA + NaCl +AVG	1.41 ± 0.13 ^g^	51.8 ± 2.97 ^f^	6.8 ± 0.76 ^f^	232 ± 14.3 ^g^	140 ± 15.2 ^g^	0.45 ± 0.03 ^g^	0.42 ± 0.019 ^g^
Eth + NaCl + Flu	2.68 ± 0.26 ^e^	106.8 ± 5.92 ^d^	15.4 ± 1.19 ^d^	312± 25.4 ^e^	238 ± 22.8 ^e^	0.79 ± 0.05 ^e^	1.13 ± 0.12 ^e^

**Table 4:** Content of sulfur, cysteine, GSH, and nitrogen, and activity of NR, APX, and GR, in mustard (*Brassica juncea* L. cv. Pusa Vijay) seedlings grown under normal and 100 mmol NaCl stress conditions at 30 days after sowing, after foliar treatment of 2.0 mmol Eth, 25 µmol ABA, 25 µmol Flu, or 0.01 µmol AVG at 15 days after sowing. Values are presented as means ± SE (n = 4). Data means followed by the same letter are not significantly different at *p* ≤ 0.05 according to the LSD test. ABA: abscisic acid; APX: ascorbate peroxidase; AVG: aminoethoxyvinylglycine; Eth: ethephon; Flu: fluoridone; GR: glutathione reductase.

Treatments	Sulfur Content	Cysteine Content	GSH Content	Nitrogen Content (mg g^−1^ Dry Mass)	NR Activity (nmol NO_2_ g^−1^ FW h^−1^)	APX Activity	GR Activity
	(mg g^−1^ Dry Mass)					(U mg^−1^ Protein min^−1^)	
Control	7.45 ± 0.46 ^f^	18.9 ± 1.41 ^g^	349 ± 21 ^g^	19.6 ± 1.21 ^d^	312 ± 11.9 ^d^	0.72 ± 0.033 ^h^	0.21 ± 0.019 ^h^
NaCl	6.10 ± 0.49 ^h^	34.2 ± 2.33 ^e^	412 ± 29 ^d^	11.5 ± 0.97 ^g^	218 ± 9.12 ^g^	1.42 ± 0.067 ^e^	0.31 ± 0.023 ^e^
Eth	11.53 ± 0.87 ^a^	45.1 ± 2.51 ^b^	442 ± 37 ^b^	28.3 ± 1.81 ^a^	412 ± 15.6 ^a^	2.23 ± 0.11 ^c^	0.49 ± 0.033 ^c^
ABA	9.32 ± 0.71 ^d^	37.2 ± 2.37 ^d^	367 ± 24 ^e^	23.2 ± 1.56 ^b^	368 ± 14.8 ^c^	1.32 ± 0.050 ^f^	0.28 ± 0.017 ^f^
Eth + NaCl	10.13 ± 1.08 ^b^	52.7 ± 2.67 ^a^	452 ± 43 ^a^	21.2 ± 1.37 ^bc^	372 ± 13.8 ^b^	2.60 ± 0.127 ^a^	0.57 ± 0.039 ^a^
ABA + NaCl	8.11 ± 0.75 ^e^	38 ± 2.32 ^c^	427 ± 34 ^c^	16.7± 1.06 ^e^	301 ± 11.6 ^e^	1.85 ± 0.061 ^d^	0.38 ± 0.031 ^d^
ABA + NaCl + AVG	6.71 ± 0.37 ^g^	31.6 ± 2.04 ^ef^	359 ± 22 ^f^	15.5 ± 1.01 ^f^	245 ± 10.1 ^f^	0.98 ± 0.041 ^g^	0.23 ± 0.022 ^g^
Eth + NaCl + Flu	9.36 ± 1.21 ^c^	51.3 ± 2.61 ^a^	451 ± 38 ^a^	20.9 ± 1.19 ^c^	369 ± 15.14 ^bc^	2.34 ± 0.116 ^b^	0.54 ± 0.035 ^b^

## Data Availability

Data are contained within the article and Supplementary File.

## Supplementary Materials

The following supporting information can be downloaded at: https://www.mdpi.com/article/10.3390/plants13162307/s1, Supplementary File S1: Methodology details.
