# Supplementary material for: Ethylene Is Crucial in Abscisic Acid-Mediated Modulation of Seed Vigor, Growth, and Photosynthesis of Salt-Treated Mustard
Source: Plants (Basel). 2024 Aug 19;13(16):2307. doi: 10.3390/plants13162307 (PMC11360230; doi:10.3390/plants13162307)
Supplement: Supplementary file 1 [file plants-13-02307-s001.zip › plants-3057997-supplementary.pdf]

Ethylene is crucial in abscisic acid-mediated modulation of seed vigor, growth and photosynthesis of salt-treated mustard

Asim Masood[1]*, Sheen Khan[1], Iqbal Mir[1], Naser A. Anjum[1], Faisal Rasheed[1], Abdulrahman Al-Hashimi[2]* and Nafees A. Khan[1]*

[1]Plant Physiology and Biochemistry Laboratory, Department of Botany, Aligarh Muslim University, 202002, Aligarh, India

[2]Department of Botany and Microbiology, College of Science, King Saud University, Riyadh 11451, Saudi Arabia

* Correspondence: naf9.amu@gmail.com, asim.bot@gmail.com, aalhashimi@ksu.edu.sa

Supplementary File S1: Methodology details

Determination of sulfur content

Oven-dried leaf powder (100 mg) was taken in digestion tube of 75 ml capacity. In digestion tube, 4.0 ml acid mixture (consists of concentrated nitric acid and perchloric acid in the ratio of 1:1) and 7.5 mg of selenium dioxide as a catalyst was added. The digestion was carried out till the digested solution became colourless. Following digestion, the volume was made up to 75.0 ml with de-ionized water. Total S in plant samples were estimated according to the turbidimetric method of Chesnin and Yien, (1951) . A 5 ml aliquot was pipette out from the digested solution for turbidity development in 25 ml volumetric flask. Turbidity was allowed to develop for 2 min. The values were recorded at 415 nm within 10 min after the turbidity development. A blank was also run simultaneously after each set of determination. The amount of sulfate was calculated with the help of a calibration curve drawn afresh using a series of K_2SO_4 solutions.

Determination of cysteine content

The content of cysteine in leaves was determined spectrophotometrically adopting the method of Giatonde (1967). Fresh leaf (0.5 g) was homogenized in 5% (w:v) ice-cold perchloric acid. The final volume of 4 ml g^{-1} of plant tissue was used. The suspension was centrifuged at 2,800 g for 1 h at 5 °C and supernatant was filtered through Whatman No. 30 paper. One ml of filtrate was treated with acid ninhydrin reagent. The extinction was read at 580 nm and the amount of cysteine was calculated with reference to a calibration curve obtained similar conditions for the amount of cysteine.

Determination of GSH content

GSH content was determined using Anderson (1985) method. To assess reduced glutathione (GSH), an enzyme recycling method was employed, wherein GSH was first oxidized by 5,5-dithiobis-2-nitrobenzoic acid (DTNB) and then reduced back by NADPH in the presence of glutathione reductase (GR). For the specific determination of oxidized glutathione (GSSG), GSH was masked by derivatization with 2-vinylpyridine. Fresh leaf tissues (500 mg) were pulverized in liquid nitrogen using a mortar and pestle and suspended in 2 mL of 5% (w/v) sulfosalicylic acid. Following centrifugation at 12,000×g for 10 minutes, a 300 μL aliquot of the supernatant was taken and neutralized by adding 18 μL of 7.5 M triethanolamine. To determine the concentrations of both GSH and GSSG, a 150 μL sample was used. Another sample underwent pretreatment with 3 μL of 2-vinylpyridine for 60 minutes at 20°C to derivatize and mask GSH, allowing for the subsequent determination of GSSG alone. In each case, 50 μL aliquots of the samples were mixed with 700 μL of 0.3 mM NADPH, 100 μL of

DTNB, and 150 μL of buffer containing 125 mM sodium phosphate and 6.3 mM EDTA at pH 6.5. Subsequently, a 10 μL aliquot of GR (5 U mL^{-1}) was added, and the change in absorbance at 412 nm was monitored at 30°C. Standard curves were prepared for GSH and GSSG, covering ranges of 5-55 nmol and 1-5 nmol, respectively.

Determination of nitrogen content

Leaf N content was estimated by the Kjeldahl digestion method as described by Lindner (1944). A 10 ml aliquot of the digested material was taken in a 50 ml volumetric flask. To this, 2 ml of 2.5 N sodium hydroxide and 1ml of 10% sodium silicate solutions were added to neutralize the excess of acid and to prevent turbidity, respectively. The volume was made up to the mark with de-ionized water. In a 10 ml graduated test tube, 5 ml aliquot of this solution was taken and 0.5 ml Nessler's reagent was added. The final volume was maintained with de-ionized water. The contents of the test tubes were allowed to stand for 5 min for maximum colour development. The optical density of the solution was read on a spectrophotometer at 525 nm.

Determination of nitrate reductase activity

The activity of nitrate reductase (EC 1.6.6.1) in leaves was measured by preparing an enzyme extract using the method of Kuo et al. (1982). Leaf tissue (1.0 g) was frozen in liquid N_2, ground to a powder with a chilled mortar and pestle, and then stored at -80 ºC. The powder was thawed for 10 min at 4 ºC and was homogenized in a blender in 250 mM Tris-HCl buffer, pH 8.5, containing 10 mM Cys, 1mM EDTA, 20μM FAD, 1 mM DTT, and 10% (v/v) glycerol. Nitrate reductase activity was assayed as the rate of nitrite production at 28 ºC. The assay mixture contained 10 mM KNO_3, 0.065 M HEPES (pH 7.0), 0.5 mM NADH in 0.04 mM phosphate buffer (pH 7.2) and enzyme in a final volume of 1.5 ml. The reaction was initiated by adding NADH. After 15 min the reaction was terminated by adding 1 ml of 1 N HCl solution containing 1% sulfanilamide followed by the addition of 1 ml of 0.02% aqueous N-1-napthylethylene-di-amine-dihydrochloride (NED). The absorbance was read at 540 nm using a spectrophotometer (SL164 Elico, New Delhi, India) after 10 min.

Measurement of ethylene evolution

For measurement of ethylene evolution, leaf material (0.5g) were cut into small pieces and was placed into 30 ml tubes containing moist paper to minimize evaporation from the tissue and were stoppered with secure rubber caps and placed in light for 2 h under the same condition used for plant growth. An earlier experiment showed that 2 h incubation time was adequate for ethylene detection without the interference of wound-induced ethylene, which began after 2 h of leaf incubation. A 1 ml gas sample from the tubes was withdrawn with a hypodermic syringe and assayed on a gas chromatograph (Nucon 5700, New Delhi, India) equipped with a 1.8 m *Porapack* N (80–100 mesh) column, a flame ionization detector, and data station. Nitrogen was used as carrier gas. The flow rates of nitrogen, hydrogen, and oxygen were 30,30 and 300 ml min^{-1}, respectively. The detector was set at 150 °C. Ethylene was identified based on the retention time and quantified by comparison with peaks from standard ethylene.

Measurement of ABA content

The content of ABA was determined by adopting the method of Hung and Kao (2003) with slight modifications. Leaves were frozen with liquid nitrogen immediately and ground into fine powder. The powder was homogenized in the extraction solution (80% methanol containing 2% glacial acetic acid). The crude extract was centrifuged and passed through polyvinylpyrrolidone column and C18 cartridges to remove plant pigments and other non-polar compounds which could interfere in the immunoassay. The eluates were then concentrated to dryness by vacuum evaporation and resuspended in Tris-buffered saline before enzyme-linked immunosorbent assay (ELISA). Afterwards, ABA was determined with ABA immunoassay detection kit (PGR-1; Sigma-Aldrich, St. Louis, MO, USA) as per

the user manual. The ABA content was estimated from a calibration curve plotted by using standard ABA and values were recorded at 405 nm.
